# Evaluation of the Method of Periodic Medium Renewal of *Bacillus aryabhattai* RAF 5 and Analysis of P(3HB) Production

**DOI:** 10.3390/polym17070968

**Published:** 2025-04-02

**Authors:** Aidana Rysbek, Urszula Jankiewicz, Ewelina Pogorzelska-Nowicka, Jarosław Wyrwisz, Sailau Abeldenov, Aleksandra M. Mirończuk, Agnieszka Richert

**Affiliations:** 1 LLP “National Center for Biotechnology”, 13/5, Kurgalzhynskoye Road, Astana 010000, Kazakhstan; abeldenov@biocenter.kz; 2 Department of Biochemistry and Microbiology, Faculty of Agriculture and Biology, Warsaw University of Life Sciences—SGGW, 02-776 Warsaw, Poland; urszula_jankiewicz@sggw.edu.pl; 3 Department of Technique and Food Product Development, Institute of Human Nutrition Sciences, Warsaw University of Life Sciences—SGGW, 02-776 Warsaw, Poland; ewelina_pogorzelska@sggw.edu.pl (E.P.-N.); jaroslaw_wyrwisz@sggw.edu.pl (J.W.); 4 Department of Biotechnology and Food Microbiology, Wroclaw University of Environmental and Life Sciences, 51-630 Wroclaw, Poland; aleksandra.mironczuk@upwr.edu.pl; 5 Institute of Biology, Laboratory for Biosustainability, Wroclaw University of Environmental and Life Sciences, 51-630 Wroclaw, Poland; 6 Department of Genetics, Faculty of Biology and Veterinary Science, Nicolaus Copernicus University, Torun, Gagarina 11, 87-100 Torun, Poland; a.richert@umk.pl

**Keywords:** *Bacillus aryabhattai*, poly(3-hydroxybutyrate) (P(3HB)), permeability of membranes, periodic medium renewal (PMR), sustainability

## Abstract

The purpose of this study was to assess the ability of *Bacillus aryabhattai* RAF 5 to produce P(3HB) under conditions of periodic medium renewal (PMR). The producer was isolated and confirmed using transmission electron microscopy (TEM), which revealed the presence of more than 10 dense P(3HB) granules per cell. The purity of the isolated polymer was evaluated using Fourier transform infrared spectroscopy (FTIR). The maximum concentration of P(3HB) reached 18.70 g/L with biomass accumulation of 21.44 g/L after 120 h of incubation under PMR conditions, which is 6.61 g/L higher than the concentration of P(3HB) obtained with the standard cultivation method. The resulting polymer was later used to create a membrane, which was then tested for permeability to water vapor, oxygen, carbon dioxide, and a biofilm puncture test. The resulting P(3HB)-based membranes have promising barrier properties, indicating their suitability for various industrial applications, including biomedical devices.

## 1. Introduction

Poly(3-hydroxybutyrate) (P(3HB)) is a biodegradable polymer that has attracted increasing attention for its possible application as an environmentally friendly substitute for petroleum-based plastics. The unique properties of P(3HB) include its mechanical strength, thermoplasticity, and biodegradability, which make it a good candidate for use in many areas. Some of the products that can be generated from P(3HB) include flexible biofilms, nonwoven materials, degradable medical sutures, semipermeable membranes, containers, and packaging materials. In addition, the compatibility and degradability of P(3HB) make it suitable for medical and diagnostic applications [1]. At present, there is an increasing focus on applying polymeric carriers for drug delivery purposes. Numerous attempts are being made to design systems that can help fight infectious diseases and oncological pathologies effectively. Polymer–drug conjugates and amphiphilic block copolymers are examples of the important subcategories of such technologies, and nanoparticle-based polyester delivery systems among them show the most encouraging results when it comes to P(3HB)-based systems and their unique physicochemical and biological properties [2].

The reported P(3HB)-synthesizing potential of bacteria is considered a promising strategy in which specific techniques can be applied to obtain a polymer with the required characteristics. More than 500 different microbial species, including the *Alcaligenes*, *Chromatium*, *Hyphomicrobium*, *Methylobacterium*, *Nocardia*, *Pseudomonas*, *Rhizobium*, *Spirillum*, and *Streptomyces* genera, have been reported to synthesize P(3HB) in substantial quantities. Among these genera, bacteria belonging to the *Bacillus*, *Alcaligenes*, *Ralstonia*, *Azotobacter*, and *Methylobacterium* genera are considered promising candidates for the natural biosynthesis of biopolymers. These species can synthesize polyesters using not only low-cost substrates, including acetate, glycerol, molasses, methanol, and glucose, but also under the constant restriction of essential mineral nutrient elements such as nitrogen and phosphorus. Moreover, these species are relatively easy to clone for genes involved in the biosynthesis of reserve polyesters. Genes encoding enzymes of PHA biosynthesis have been cloned, and recombinant strains of *Escherichia coli* with a functionally active complex of these genes have been obtained [3,4,5].

Under unfavorable environmental conditions, that is, when the cell is exposed to various stressful factors, such as temperature changes, exposure to ultraviolet light, and limitation of trace elements and macronutrients (such as nitrogen, phosphorus, or oxygen), in the presence of an excess of an exogenous carbon source to more accurately describe the conditions promoting PHA biosynthesis, bacterial species, particularly *Bacillus* strains, can accumulate P(3HB) in the form of carbon and energy storage material [6].

Recent studies have shown that the strain MAPCS4 *Priestia megaterium* (*Bacillus megaterium* antiquated since 2020 [7]), isolated from gasoline-contaminated soil, demonstrates hyperproduction of P(3HB). Under optimized fermentation conditions in a modified mineral salt medium with cream apple hydrolysate (5% *v*/*v*), P(3HB) production reaches up to 7.92 g/L [8]. *Priestia megaterium* LVN01, discovered in Colombia, is capable of processing industrial byproducts such as ficus juice, sugarcane molasses, and residual glycerol to produce poly(3-hydroxybutyrate) (P(3HB)). Periodic aerobic fermentation leads to a maximum cell dry weight of 0.56 g/L after 60 h, while the highest P(3HB) yield reaches 360 mg/L after 16 h [9]. These results highlight the potential of *Bacillus megaterium* as an effective microorganism for biotechnological processes aimed at P(3HB) production, including the use of waste and byproducts.

*Priestia megaterium* and *Bacillus aryabhattai* are the most closely related strains [10]. In earlier studies, *Bacillus aryabhattai* was investigated as a potential source of biosurfactants [11], but there are fewer reports in which *B. aryabhattai* is considered a potential producer of P(3HB).

For large-scale industrial applications, further research is required to optimize the biosynthesis process of P(3HB) by *Bacillus aryabhattai* strains to enhance productivity. In this study, subsequent optimization of the synthesis conditions and characterization of P(3HB) produced by the *Bacillus aryabhattai* RAF 5 strain, previously isolated from chestnut soils in northern Kazakhstan, were conducted [12]. The aim of the study was to comprehensively investigate the ability of the *Bacillus aryabhattai* RAF 5 strain to accumulate P(3HB), analyze its growth under batch cultivation conditions, and examine the properties of membranes fabricated from bacterial P(3HB). Particular attention was given to evaluating membrane characteristics, including permeability to water vapor, oxygen, and carbon dioxide.

## 2. Materials and Methods

### 2.1. Microorganism

The *Bacillus aryabhattai* RAF 5 strain was isolated from chestnut soils in Astana, Kazakhstan, at the National Center for Biotechnology, Laboratory of Environmental Biotechnology. The strain was routinely maintained on the Luria–Bertani medium (LB: 10 g/L peptone, 10 g/L NaCl, 5 g/L yeast extract, 17 g/L agar). For long-term storage, microorganisms were maintained at −80 °C in a glycerol solution (50% glycerol and 50% distilled water). Physiobiochemical properties of the strain, including growth dependence on temperature and pH, as well as its physical characteristics, were determined using previously described methods [13,14]. Unless otherwise specified, all experiments were conducted at 37 °C. The strain has been deposited in the Collection of PHA Producers at the Depository of the National Center for Biotechnology in Astana, classified as biosafety level 4, under the accession number IMD-B-462.

### 2.2. Culture Media and Growing Conditions

To achieve stable and sustained biosynthesis of P(3HB), a periodic medium renewal (PMR) method was employed. Cultivation was conducted in a 4 L mineral medium in Erlenmeyer flasks with a total volume of 10 L, with periodic renewal every 24 h. Half of the medium volume was removed and replaced with 2 L of fresh medium containing 0.3 g/L nitrogen, which stimulated cell growth. Strain *Bacillus aryabhattai* RAF 5 was grown in a mineral medium: 20 g/L glucose, 1.0 g/L (NH_4_)_2_SO_4_, 4.35 g/L Na_2_HPO_4_, 1.3 g/L KH_2_PO_4_, 0.2 g/L KCl, 0.02 g/L MgCl_2_, 0.001 g/L CaCl_2_, 0.01 g/L FeCl_3_, 0.001 g/L MnCl_2_, and 0.1 g/L Na_2_SO_4_. The volume of the medium was adjusted to 1 L, and the pH was adjusted to 7.0 using HCl. The culture was maintained at 37 °C with aeration on a shaker at 150 rpm.

Statistical analysis of the results was performed using conventional methods, with the standard software package of Microsoft Excel, with three repetitions.

### 2.3. TEM Analysis of the Isolate

The agar culture of microorganisms was suspended in 2 mL of a 5% glutaraldehyde solution (pH 8.0) until turbidity reached 10 standard units. Incubation was carried out at 4 °C for 2 h.

To prepare ultrathin sections for Transmission electron microscopy (JEM 1400 Plus, Beaverton, OR, USA) examination, the material was suspended in 500 µL (depending on the volume of the capsule) of a filling mixture with a catalyst and transferred to beam capsules. Polymerization of the samples was carried out at a temperature of 56 °C for 24 h. After polymerization of the resin, the solidified blocks were removed from the capsule. The block was fixed in the holder with the top of the pyramid facing up, and the holder was placed in the microtome clip. The blocks were sharpened, and ultrathin sections were prepared. The slices were mounted on grids with carbon formvar films. The sections were contrasted with a saturated 1% solution of uranyl acetate in 70% ethanol at a temperature of 56 °C for 5–10 min or with a 1% aqueous solution of uranyl acetate at room temperature for 1 h. For this purpose, a grid with a downward-facing section was lowered onto the surface of the contrast agent in a Petri dish. After contrasting, the grids were taken with tweezers, immersed in distilled water for rinsing, dried on filter paper, and transferred to a Petri dish for TEM examination [15].

### 2.4. Extraction and Quantitative Determination of P(3HB) Using a Chemical Method

The method described by Lowe [16] was used to obtain P(3HB) films and calibrate the fluorescence method. The isolates were cultured in a modified Law and Slepecky (LS) mineral medium at 37 °C for 48 h in a shaker at 160 rpm. A 2 mL aliquot of the bacterial suspension (if A600 ≥ 0.2) was transferred to microtubules and centrifuged for 10 min at 6000 rpm (centrifuge 5415 D, Enfield, CT, USA). The granule was resuspended in 5 mL of sterile water and subjected to ultrasound treatment for 5 min.

A 2 mL portion of the suspension was mixed with 2 mL of 2 N HCl and heated in a water bath at 100 °C for 2 h, followed by centrifugation at 6000 rpm (Centrifuge 5415 D, Eppendorf, Enfield, CT, USA) for 10 min. The resulting granules were dried, suspended in 5 mL of chloroform, tightly sealed, and shaken overnight at 150 rpm at a temperature of 28 °C. The tubes were then centrifuged for 20 min at 6000 rpm, and a 0.1 mL chloroform phase was extracted.

The transparent, chloroform-containing bottom fraction was poured into a glass vial and air-dried. A white precipitate (P(3HB)) formed at the bottom of the vial, to which 1 mL of concentrated H_2_SO_4_ was added and heated at 100 °C for 20 min. Hydrolysis and dehydration led to the formation of crotonic acid, resulting in a marsh-colored solution. The sample was then analyzed using a spectrophotometer.

To measure absorbance using a spectrophotometer, the samples were hydrolyzed with 5 N NaOH, and the pH was adjusted to 4.0. Then, the samples were filtered through a membrane filter with a pore size of 0.45 microns. The absorbance of the solution was measured using a spectrophotometer (GE GeneQuant 100, Great Britain) at a wavelength of 235 nm, corresponding to the maximum absorption of crotonic acid. A neutralized sulfuric acid solution was used as a control sample, and the results were compared with a standard P(3HB) sample (Sigma Aldrich, Rockville, MD, USA). The amount of P(3HB) was calculated using the following formula:

[P(3HB)] (mg) = 0.0056 × A_235_

where A_235_ represents the absorbance of the solution at 235 nm [17].

### 2.5. Extraction of P(3HB) from Biomass

The production process of P(3HB) was assessed using the method described by Law and Slepecky [16], with polymer quantification based on a standard curve constructed using a commercial P(3HB) sample (Sigma-Aldrich, Rockville, MD, USA). The bacterial culture, grown in a mineral medium with periodic renewal, was centrifuged at 10,000 rpm at 4 °C for 10 min to separate the pellet. The pellet was washed with an equal volume of acetone and ethanol (50 mL per 10 g of biomass) to remove impurities. The biomass was then resuspended in an equal volume of a 4% sodium hypochlorite solution and incubated at room temperature for 30 min.

After treatment, the mixture was centrifuged again (10,000 rpm, 10 min) to precipitate lipid granules. The supernatant was discarded, and the pellet was repeatedly washed with acetone and ethanol. The resulting granular polymer was dissolved in hot chloroform and filtered through filter paper with a pore size of 110 µm, pretreated with hot chloroform. To obtain the purified product, the solution was dried under a fume hood overnight.

### 2.6. IR-Fourier Spectroscopy to Determine the Characteristics of Extracted P(3HB)

The chemical nature of the extracted polymer was confirmed by the method of infrared spectroscopy with inverse Fourier transform using the protocol we described previously [18]. Briefly, an aliquot of 1 g P(3HB) extracted from RAF 5 was analyzed using an IR Fourier spectrometer (Nicolet IS 10, Thermo Fisher Scientific, Waltham, MA, USA). The spectra were recorded in the range of 400–4000 cm^−1^. The P(3HB) spectra from Sigma-Aldrich, Rockville, MD, USA, were used as a standard for comparison.

### 2.7. Preparation of a P(3HB) Membrane

The examined membrane was prepared using the solvent-casting method. P(3HB) was dissolved in chloroform to obtain a 3% (*w*/*v*) polymer solution. To obtain a P(3HB) membrane, 50 mL of the prepared mixture were poured into Petri glass dishes (14.5 cm in diameter) and left for 3 days to form a polymer film [19,20,21]. The thickness of the films was measured using an electronic thickness gauge (0.001/0–12.7 mm). The thickness ranged from 0.075 to 0.080 mm.

### 2.8. Mechanical Properties

#### 2.8.1. Permeability of Water Vapor

The water vapor permeability (Pv) was determined in accordance with PN-EN ISO 15106-1 “Plastics-Films and boards-Determination of Water Vapor Transmission Rate-Part 1: Moisture sensor Method” using an L80-5000 type apparatus (PBI Dansensor, USA) [22]. This test determines the amount [g] of water vapor permeating through a given surface of the sample per unit time and at constant temperature. Five measurements were made for each sample, and the arithmetic mean of these measurements was taken as the test result. The test was carried out at a temperature of 38 °C.

#### 2.8.2. Permeability of Oxygen

Determination of oxygen permeability (PO) was carried out according to ASTM F 1927-98 “Standard Test Method for Determination of Oxygen Gas Transmission Rate, Permeability and Permeance at Controlled Relative Humidity Through Barrier Materials Using a Coulometric Detector” using a MultiPerm O_2_-CO_2_ DC test stand (PermTech srl, No. B02Y0, UK) [23]. Three measurements were made for each sample, and the arithmetic mean of the three measurements was taken as the test result. The tests were carried out using a mask with a measuring area of 2.01 cm^2^. The obtained measurement results correspond to the thickness of the test sample.

#### 2.8.3. Permeability of Carbon Dioxide

Carbon dioxide permeability (PD) was determined according to ASTM F2476-20 “Standard Test Method for the Determination of Carbon Dioxide Gas Transmission Rate (CO_2_TR) Through Barrier Materials Using an Infrared Detector” using a MultiPerm O_2_-CO_2_ DC test stand (PermTech srl, No. B02Y0, UK) [24]. The obtained measurement results correspond to the thickness of the test sample.

#### 2.8.4. Puncture Test

The puncture test was performed using an Instron 5965 universal testing machine (Norwood, MA, USA). The maximum force required to puncture the P(3HB) samples served as a key parameter for assessing textural properties. Hardness, defined as the force at fracture (N), was evaluated in P(3HB) films (3% solution) with a thickness of 0.22 µm and a surface area of 3.38 cm^2^ (13 mm wide and 26 mm long). The biopolymer sample was secured between two steel plates, and a cylindrical 8 mm diameter probe was applied at the center. The test was conducted at a crosshead speed of 60 mm·min^−1^ using a 50 N load cell, continuing until the probe fully penetrated the P(3HB) sample. Two samples were tested.

## 3. Results

### 3.1. Physiological and Biochemical Factors on Growth and P(3HB) Production

**Figure 1:**
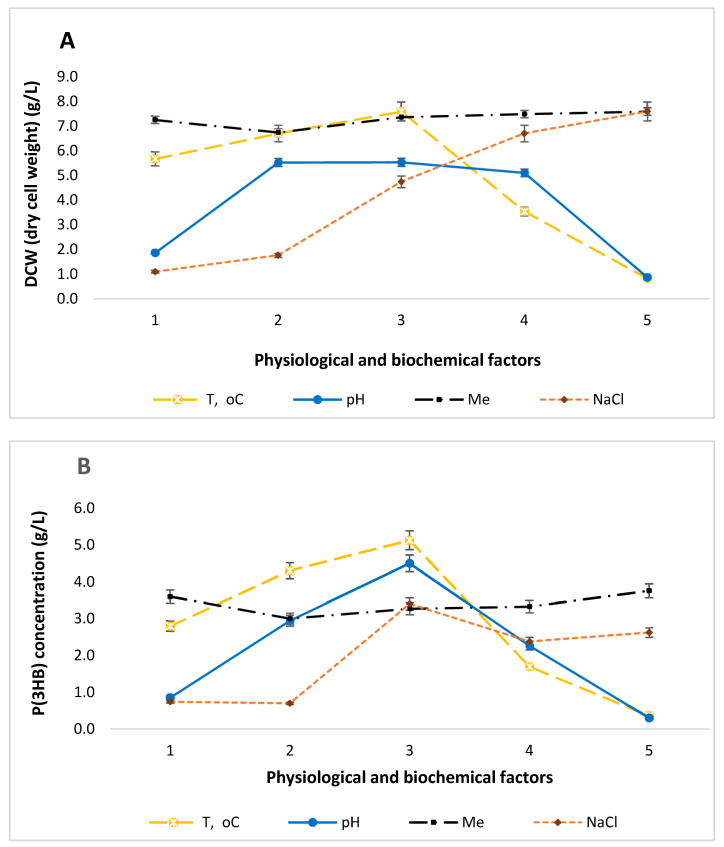
Influence of physiological and biochemical factors on the growth of *Bacillus aryabhattai* RAF 5 cells (**A**) and P(3HB) content (**B**).

The *Bacillus aryabhattai* RAF 5 isolate was cultivated in a mineral medium. On agar plates, this strain formed round, beige colonies with a diameter of 2 mm, which were slightly convex with smooth edges. The colony consistency was viscous, and the surface was smooth. The optimal growth temperature for *B. aryabhattai* RAF 5 was 37 °C, with an optimal pH of 7.0. No pigment production was observed in the nutrient medium. The biological titer of the strain reached 10^9^ CFU/mL. In Figure 1, we presented the effect of physiological and biochemical factors on cell growth of the *Bacillus aryabhatai* RAF 5 strain (A) and the content of P(3HB) (B). The values from 1 to 5 correspond to the following parameters: temperature (°C): 20, 30, 37, 40, and 45 °C; pH: 5.0, 6.0, 7.0, 8.0, and 9.0; metal ions at a concentration of 0.3 g/L: CoCl_2_, CuSO_4_, FeCl_3_, MnCl_2_, and a control without metals; NaCl concentration: 10%, 8%, 4%, 2%, and a control (0%). Measurements were taken over a 24 h period.

Temperature-dependent patterns of P(3HB) content were observed for the *Bacillus aryabhattai* RAF 5 strain, with the minimum and maximum P(3HB) concentrations recorded at 20 °C, 30 °C, 37 °C, 40 °C, and 45 °C over a 24 h period. Notably, P(3HB) synthesis was completely absent at 45 °C [25]. While pH fluctuations in the range of 6.0–8.0 had no significant effect on the growth of *B. aryabhattai* RAF 5 or the synthesis of alkanoates [26]. The lowest P(3HB) production was observed at pH 5.0 and 45 °C, whereas the maximum P(3HB) concentration was achieved at pH 7.0 and 37 °C. The strain exhibits some resistance to the presence of heavy metals, as its growth levels remain comparable to the control values (without metals). The data indicate that the growth of strain RAF 5 decreases with increasing NaCl concentration. The highest P(3HB) production for this strain was observed at a salt concentration of 2% [27].

*Bacillus* strains isolated in [28] also exhibited the highest P(3HB) synthesis at pH 7.0, consistent with findings from [29], with an optimal temperature of 37 °C. In [30], within the pH range of 6.5–9.0 and temperature range of 25–45 °C, the maximum P(3HB) concentration was recorded at pH 8.0 and 35 °C.

Overall, ecological and physiological incubation conditions, such as pH and temperature, are crucial not only for sustaining bacterial growth, but also for achieving the highest possible P(3HB) concentration. These factors are of particular interest at both the laboratory and industrial scales for optimizing production volumes.

### 3.2. Effect of Periodic Medium Renewal on Biomass Growth and P(3HB) Production

During the experiments, the frequency of medium renewal and the duration of cultivation cycles were varied, with each cycle conducted in a periodic mode. The following parameters were investigated: removal and replacement of 50% of the medium with a fresh mineral medium. These volumes were selected to ensure that the culture was enriched with essential growth components for new cell formation while maintaining a sufficient amount of active biomass capable of synthesizing P(3HB).

**Figure 2:**
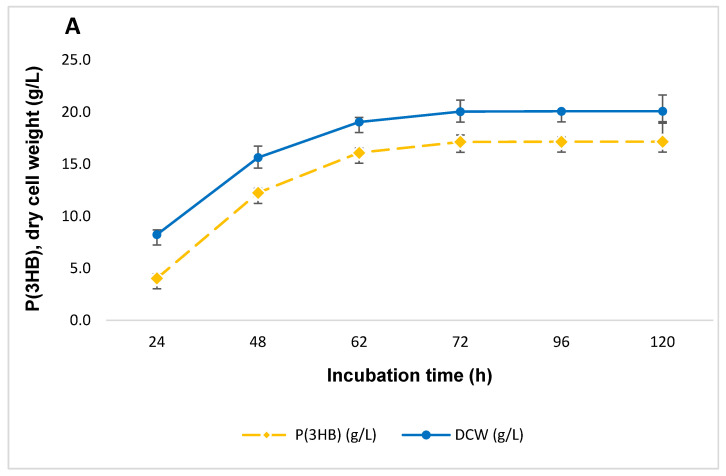

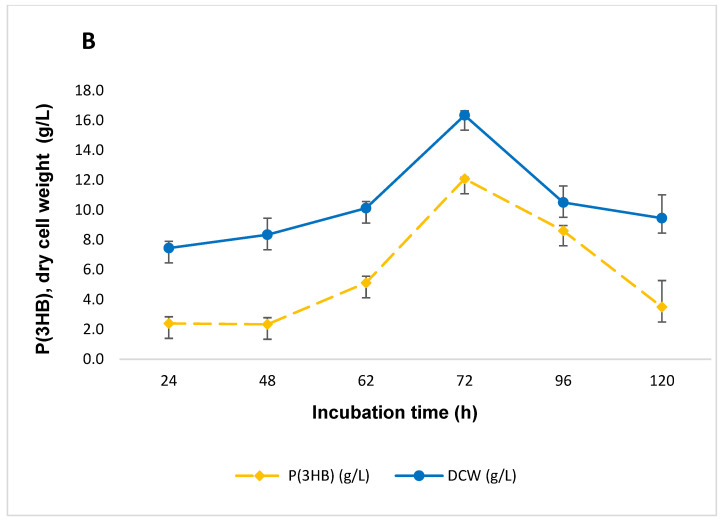
Biomass content and P(3HB) concentration with periodic medium renewal (**A**) and in the standard mode of cultivation (**B**) in a nutrient broth (NB) medium, strain *B. aryabhattai* RAF 5. DCW—dry cell weight; P(3HB)—poly(3-hydroxybutyrate).

Figure 2 presents the results of periodic cultivation. The biosynthesis process in this model remained intensive for 120 h (5 days), with a high P(3HB) concentration reaching 18.7 g/L by the 120th hour.

The P(3HB) concentration was 18.70 g/L, with biomass accumulation of 21.44 g/L after 120 h when periodic additions of a mineral medium were applied. Under the standard cultivation conditions, the maximum P(3HB) concentration was 12.09 g/L at 72 h, while biomass accumulation reached 16.34 g/L.

This method has been successfully employed in the production of citric acid from ethanol [31], glucose [32], and glycerol-containing waste [33,34], as well as when using glucose as the sole carbon source [6].

### 3.3. TEM Analysis of Selected Isolates of Bacteria Producing P(3HB)

Transmission electron microscopy (TEM) analysis revealed the accumulation of dense P(3HB) granules in the cytosol of the isolate. The cytosol was observed to be filled with large granules, which appeared either individually or in early-stage formation. The TEM images support the quantitative findings, confirming that the RAF 5 strain is an efficient P(3HB) producer.

**Figure 3:**
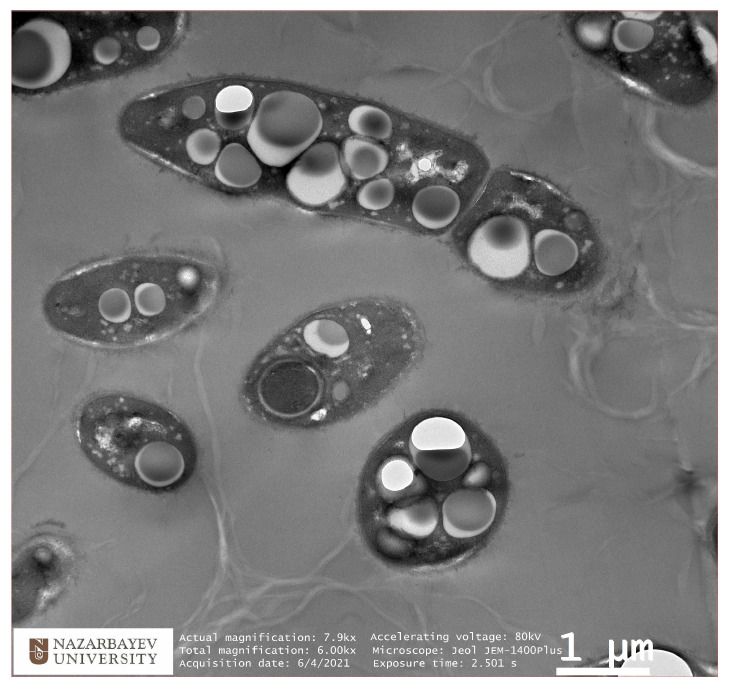
Image of cells producing P(3HB) after 20 h of incubation: half-day cultures of RAF 5. White granules inside bacterial cells depict P(3HB) granules accumulated inside them.

Figure 3 shows an image of a TEM RAF 5 isolate with a high P(3HB) content.

The size and number of cells varied, with approximately 4–13 granules per cell, each having a diameter range of 0.2–1.0 μm. Our results are in accordance with the previously reported studies [35,36].

### 3.4. Extracted P(3HB) Film from the B. aryabhattai RAF 5 Strain

**Figure 4:**
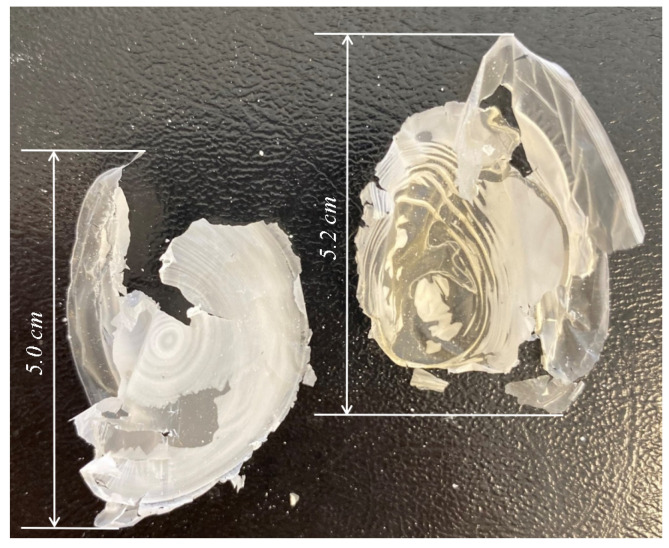
Extracted P(3HB) film from the *B. aryabhattai* strain RAF 5.

After extracting P(3HB) from the biomass, the solution was applied to clean glass Petri dishes. Following overnight drying under a fume hood, a film formed, which could be easily peeled off the surface of the dishes, as shown in Figure 4.

Thermal analysis of the P(3HB) samples was conducted using a melting point ap-paratus (Fisher-Johns, Melting Point Apparatus, USA). The melting temperature (T_melt_) of the P(3HB) samples obtained from Sigma-Aldrich and the film extracted from the RAF 5 bacteria showed similar values of T_melt_ = 175 °C, which may indicate a high molecular weight of the isolated polymer. Similar to our results, the melting temperature of the P(3HB) synthesized by the *Hydrogenophaga* sp. UMI-18 strain was also 175 °C [37,38].

### 3.5. Characteristics of FTIR

**Figure 5:**
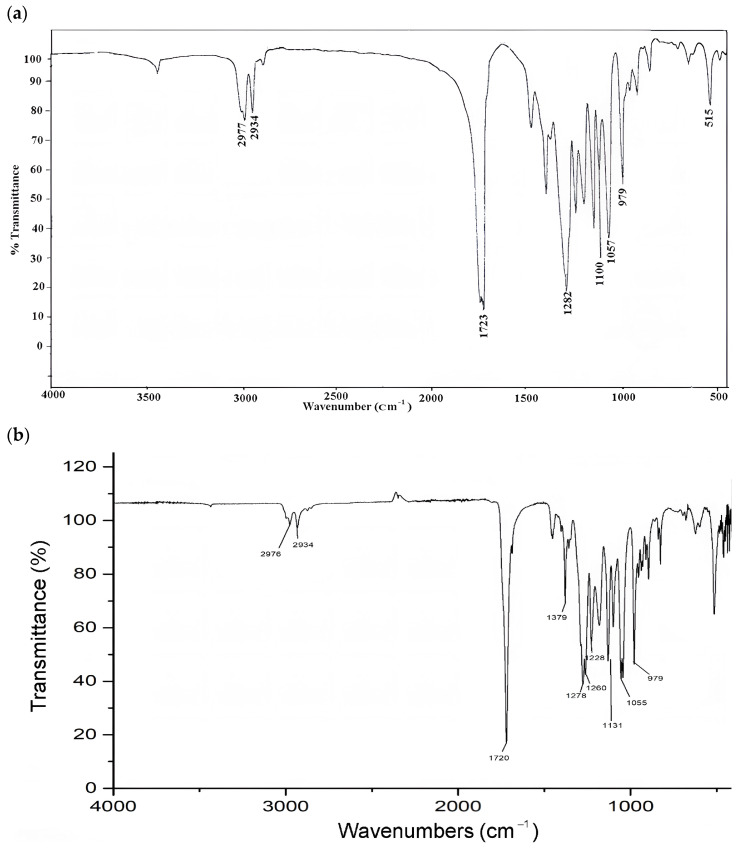
(**a**) Standard scl-PHA (PHB) [39]; (**b**) FTIR characteristics of the P(3HB) obtained from *Bacillus aryabhattai* RAF 5.

To confirm the chemical nature of P(3HB), FTIR spectra were used to determine the presence of functional groups in P(3HB) (Figure 5).

Peaks corresponding to ester, methylene, and terminal hydroxyl groups typically indicate the polymeric structure of P(3HB). It is well-known that the exact position and intensity of these peaks depend on the polymer chain length, concentration, and degree of P(3HB) crystallinity.

The characteristic band at 2976 cm^−1^ indicates the presence of asymmetric and symmetric stretching vibrations of –CH (alkane) bonds in the −CH_3_ and –CH_2_ groups, which are typically found in pure P(3HB) [40,41]. Thus, the FTIR analysis results not only closely matched the characteristics of the P(3HB) standard, but also demonstrated a high degree of purity, as confirmed by the identification of peaks corresponding to the extracted P(3HB).

### 3.6. Permeability of Water Vapor and Gases from the Prepared Membrane

The results of the research on the permeability of water vapor and gases, oxygen, carbon dioxide, and nitrogen, are presented in Table 1.

The water vapor and gas permeability of the obtained membrane are consistent with the results reported by other researchers investigating the barrier properties of membranes. The obtained parameters confirm the favorable barrier characteristics of the P(3HB) membrane [42].

The surface properties of biomaterials, particularly hydrophilicity, influence cell adhesion to materials. These mechanical parameters open up prospects for the application of P(3HB) in composite materials with other polymers for tissue engineering [43,44,45].

### 3.7. Puncture Test of Biopolymer

**Figure 6:**
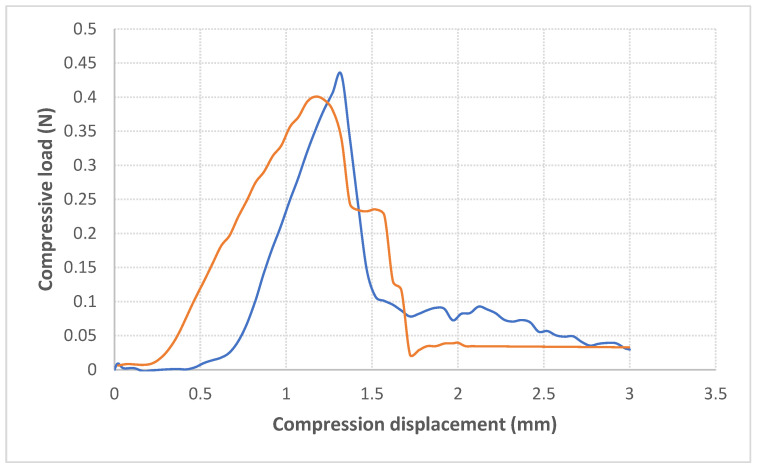
Puncture deformation test of the P(3HB) films.

For the puncture test, a biopolymer based on poly(3-hydroxybutyrate) (concentration, 3%) was dissolved in chloroform and poured into glass molds to form a film. After evaporation of the solvent and formation of the film material, the sample tended to curl. To smoothen the surface, the film was carefully moistened with water, smoothed between layers of blotting paper, and lightly dried. Immediately after that, the sample was subjected to a mechanical puncture test. Two samples, obtained using the same method, were tested, and they are represented in the Figure 6 by the red and blue lines.

Hardness, expressed as the force at fracture [N], was very similar across the tested samples, with an average value of 0.415 N. These low values indicate the high brittleness of P(3HB). Therefore, it is suggested that P(3HB) should be used in blends with other substances to improve its mechanical strength. In such a blend, P(3HB) has potential for use as biodegradable packaging and can be considered an environmentally friendly alternative to traditional polymer materials.

## 4. Discussion

This paper describes the evaluation of a cultivation method using periodic medium renewal (PMR) to achieve high concentrations of both biomass and poly(3-hydroxybutyrate) (P(3HB) production. The PMR method reduces the cost of raw materials, heat, and electricity by reducing the number of inoculations needed and increasing the time between preparation cycles. It is assumed that the presence of a substrate, operational management, and special conditions make this polymer commercially competitive compared to some plastics of fossil origin. This method provided high biosynthetic activity of the RAF 5 strain, reaching a concentration of P(3HB) of 18.70 g/L after 120 h, and the biomass reached 21.44 g/L over the same period with periodic application of a mineral medium. In the standard NB culture, the concentration of P(3HB) for RAF 5 reached 12.09 g/L after 120 h. In comparison, in the work of Aneesh Balakrishna Pillai et al. (2017), it was reported that *B. aryabhattai* PHB10 had a maximum polymer accumulation of 3.264 g/L when 20 g/L of glucose were added to the medium [46]. Similar results were reported by Balakrishna Pillai (2020), who observed that the *B. aryabhatta* strain produced 2.8 g/L of P(3HB), which is equivalent to 71.15% of the dry weight of cells in a medium with propionic acid, after 48 h incubation [47]. Although these values are not the highest described in the literature, in an article by Chonasit et al. (2014), *B. aryabhattai* ST1C produced a maximum P(3HB) content and biomass concentration of 72.31% dry cell weight (DCW) and 7.24 g/L, respectively, during a 24 h cultivation period by batch fermentation [48]. Our method is cost-effective and easy to use, which contributes to the development of more sustainable biopolymer production processes.

Currently, many bacterial strains are used for the synthesis of P(3HB), each of which has its own growth characteristics. Physicochemical factors such as pH and temperature play a crucial role in the efficiency of the synthesis of P(3HB), and optimization of these parameters has allowed increasing the concentration of the polymer. This study highlights the importance of optimizing cultivation conditions, including temperature, pH, and nutrient availability, to enhance the synthesis of P(3HB) by *Bacillus* strains. Careful control of these parameters significantly increases polymer concentration and production efficiency.

Transmission electron microscopy (TEM) analysis confirmed the accumulation of dense P(3HB) granules in the cytosol of *B. aryabhattai* RAF 5 cells, with more than 10 granules observed in each cell. These results are consistent with the results of quantitative studies, which once again confirms the effectiveness of *B. aryabhattai* RAF 5 as an effective producer of P(3HB). The chemical nature of P(3HB) was further confirmed by Fourier transform infrared spectroscopy (FTIR), which confirmed the purity of the polymer and its structural integrity.

In addition, the Law and Slepecky environment was modified with the addition of ammonium sulfate (NH_4_)_2_SO_4_ to ensure balanced growth and production of P(3HB). It was determined that the optimal concentration of (NH_4_)_2_SO_4_ is 0.3 g/L, which increases the suitability of the medium for the synthesis of P(3HB).

The concept presented in this study demonstrates promising potential for the industrial production of poly(3-hydroxybutyrate). Various analyses have confirmed the high quality of the selected substrate and the practicality of the polymer preparation strategy. This method has significant potential to be expanded to pilot production in order to assess its technical and economic feasibility.

In addition, a membrane was made from isolated P(3HB) and tested for permeability to water vapor and gases (oxygen and carbon dioxide). The obtained force value during the puncture test of the biopolymer was relatively low (0.4 N). Similarly low hardness values have been documented by Parra et al. [49], who observed that P(3HB) blended with 2% polyethylene glycol (PEG) exhibited a puncture resistance of approximately 1.5 N, which is still higher than that in our study. This difference was most likely due to the fact that polyethylene glycol acts as a plasticizer, increasing the flexibility of the material and significantly affecting the mechanical properties of the resulting blend, including its puncture resistance. Therefore, the lower force at fracture [N] observed in our study can be attributed to the absence of any plasticizer in the tested sample. Pure P(3HB) is a brittle material, and its resistance increases after being blended with a plasticizer such as PEG, as demonstrated in the cited study. These findings highlight that P(3HB) can be successfully combined with other substances, which may impart desirable and unique properties tailored to the specific type of material one aims to develop. The membrane’s favorable barrier properties suggest its potential application in the biomedical industry, highlighting both the commercial and environmental benefits of producing P(3HB).

## 5. Patents

Aipova, R., Abeldenov, S., Kurmanbayev, A., Ramankulov, Y., Rysbek, A. (2022). Strain of bacterial genus *Bacillus aryabhattai* RAF 5—producer of polyhydroxybutyrate used for the development of a biofilm. No. 7433 RK 2022/0550 (in Kazakh).

## Figures and Tables

**Table 1:** Permeability of water vapor and gases through the membrane.

Sample	Pv, g/m^2^·24 h	Po, mL/m^2^·24 h	P_D_, mL/m^2^·24 h
P(3HB)	57 ± 0.50	140 ± 0.30	705 ± 0.30

## Data Availability

The data presented in this study are available upon request from the corresponding author.
